# 16S rDNA Sequencing for Bacterial Identification in Preterm Infants with Suspected Early-Onset Neonatal Sepsis

**DOI:** 10.3390/tropicalmed9070152

**Published:** 2024-07-06

**Authors:** Sergio Agudelo-Pérez, A. Melissa Moreno, Juliana Martínez-Garro, Jorge Salazar, Ruth Lopez, Mateo Perdigón, Ronald Peláez

**Affiliations:** 1 Department of Pediatrics, Faculty of Medicine, Universidad de La Sabana, Chía 025001, Colombia; mateopebe@unisabana.edu.co; 2 Faculty of Science and Biotechnology, Universidad CES, Medellin 050022, Colombia; anrobledo@uces.edu.co (A.M.M.); jmartinezg@www.ces.edu.co (J.M.-G.); 3 Research Center, Grupo de Estudio de Enfermedades Infecciosas y Crónicas (GEINCRO), San Martin University Foundation, Sabaneta 055450, Colombia; jorge.salazarf@sanmartin.edu.co; 4 Neonatal Unit, Hospital Meissen, Bogotá 111711, Colombia; referente.neonatos@subredsur.gov.co; 5 Graduate School, Universidad CES, Medellin 050022, Colombia; rpelaezp@ces.edu.co

**Keywords:** early-onset neonatal sepsis, preterm infant, Sanger sequencing, 16S ribosomal DNA gene, antimicrobial stewardship

## Abstract

Background: The high prevalence of suspected early-onset neonatal sepsis among preterm infants leads to immediate antibiotic administration upon admission. Notably, most blood cultures for suspected early-onset neonatal sepsis do not yield a causative pathogen. This study aimed to assess polymerase chain reaction (PCR) targeting the variable region V4 of the 16S ribosomal gene (16S rDNA) and Sanger sequencing for bacterial identification in preterm infants with suspected early-onset neonatal sepsis. Methods: Therefore, this prospective study was conducted. Preterm infants with suspected early-onset neonatal sepsis were included in this study. The three groups were formed based on the risk of infection and clinical sepsis. Blood samples were collected upon admission to the neonatal unit for culture and molecular analysis. PCR amplification and subsequent Sanger sequencing of the V4 region of the 16S rDNA were performed. Results: Twenty-eight patients were included in this study. Blood cultures were negative in 100% of the patients. Amplification and sequencing of the V4 region identified bacterial genera in 19 patients across distinct groups. The predominant taxonomically identified genus was Pseudomonas. Conclusions: Amplifying the 16S rDNA variable region through PCR and subsequent Sanger sequencing in preterm neonates with suspected early-onset neonatal sepsis can enhance the identification of microbial species that cause infection, especially in negative cultures.

## 1. Introduction

Neonatal sepsis is the primary cause of death and illness among preterm infants [1]. It is categorized as early-onset sepsis if it occurs within the first 72 h of life and as late-onset sepsis if it develops 72 h after birth [2].

In contrast, suspected early-onset neonatal sepsis is a common diagnosis in preterm infants. The incidence is 166 per 1000 live births; in contrast, the incidence of early-onset culture-confirmed sepsis is 46 per 1000 live births [3]. It consists of the presence of perinatal risk factors for infection in the mother, whether associated with clinical infection in the first 72 h of life [4], and is the leading cause of antibiotic formulation in preterm infants [5].

However, approximately 95% of preterm infants treated with antibiotics for suspected early-onset neonatal sepsis have no confirmation of infection by microbiological culture [6]. The situation described previously results in the needless administration of antibiotics to preterm infants, which in turn increases the risk of illness and death [7].

However, discerning the requirement for antibiotics in suspected early-onset neonatal sepsis presents several challenges. First, there is no consensus regarding the criteria for its definition [3]. The perinatal risk factors for infection are not reliable for this purpose [4]. The clinical manifestations are nonspecific, unreliable, and overlap with those of other diseases [6]. Neonatal sepsis was confirmed by isolating microorganisms from blood cultures [8]. However, they have low sensitivity and specificity for the following reasons: presence of bacteremia due to bacteria that do not grow in traditional cultures, use of previous antibiotics in the mother, low volume of bacteremia in neonates, and prolonged time to obtain results [9,10] his has increased the use of unnecessary broad-spectrum antibiotics in preterm infants [2].

Therefore, it is essential to develop diagnostic techniques that enable the precise, impartial, and swift identification of bacteria in neonates with suspected early-onset sepsis. The current biomarkers used to determine whether to initiate antibiotics in this scenario have limitations and are unsuitable for this purpose [11]. Polymerase chain reaction (PCR) targeting specific variable regions of 16S rDNA and broad-range sequencing have been shown to be fast and effective alternatives for identifying bacteria in adult and pediatric patients [12,13,14].

The 16S rDNA gene contains both the conserved and variable regions. Analysis of these variable regions allows for differentiation between bacterial species and, in some cases, between genera, establishing them as reliable markers in phylogenetic and taxonomic studies. This makes its findings applicable in various research contexts and clinically useful for the reliable identification of pathogens. In addition, extensive 16S rDNA sequence databases have facilitated the comparison and classification of new sequences [15,16]. However, studies of neonatal populations with suspected early neonatal sepsis are limited. In this scenario, PCR of the variable regions of 16S rDNA could be used as a complementary diagnostic test for bacterial identification in preterm infants with suspected early-onset neonatal sepsis.

This study aimed to identify bacteria in the blood of preterm infants with suspected early-onset neonatal sepsis by amplification and Sanger sequencing of the V4 region of the 16S rDNA gene.

## 2. Materials and Methods

A prospective observational study was conducted on preterm infants (Ballard < 37 weeks) with a birth weight ≤ 1800 g and suspected early-onset neonatal sepsis who were admitted to the neonatal intensive care unit of the Hospital Meissen in Bogotá between March 2022 and March 2023. This study was approved by the Ethics Committee of the Universidad de La Sabana (N°100, 21 June 2021) and Hospital Meissen (register-185). Informed consent was obtained from parents for newborn admission.

Patients were included immediately upon admission to the neonatal intensive care unit after stabilization at birth in an adaptation room. Suspected early-onset neonatal sepsis was defined as the presence of perinatal risk factors for neonatal and/or clinical infection within the first 72 h. The criteria for suspected early-onset neonatal sepsis and clinical sepsis are presented in Table 1.

Neonates with perinatal asphyxia, congenital anomalies, suspected (or confirmed) metabolic disease or inborn error of metabolism, a need for transfusion of blood products in the adaptation room, and/or before taking samples for study and admission to the neonatal unit referred from another institution, and/or older than two hours of life upon admission to the neonatal unit, were excluded.

A group of preterm infants with a birth weight of less than 1800 g and without risk factors for infection immediately after birth was also included as a control.

For data analysis, three groups of patients were formed: Group 1, patients without suspected early-onset sepsis; Group 2, patients with suspected early neonatal infection and no clinical signs of early neonatal infection; and Group 3, patients with suspected early neonatal infection and clinical infection in the first 72 h of life (clinical sepsis). The primary outcome was the identification of genetic material in the blood by PCR V4 16S rDNA amplification and Sanger sequencing.

### 2.1. Blood Culture Samples

Two blood culture samples were obtained immediately upon admission to the neonatal unit under strict asepsis and antisepsis measures and were analyzed in the hospital’s microbiology department. For each sample, 1 mL of blood was taken from the newborn and injected directly into a bottle of BACT/ALERT^®^ PF PLUS blood culture for aerobes and another for anaerobes, incubated in the equipment at 37 °C for five days.

### 2.2. Blood Samples & Clinical Data

During the collection of blood cultures, an additional one (1) mL of blood was collected in an EDTA tube for molecular testing. Initially, the sample was frozen in the neonatal unit at −20 °C and then transferred, maintaining the cold chain, to the laboratories of the University of La Sabana and CES University, where it was kept frozen at −80 °C until its analysis.

Similarly, clinical variables were prospectively collected from the standard monitoring of vital signs in the neonatal unit and the results of the laboratories in the first 72 h of life, including blood count, C-reactive protein, procalcitonin, and other clinical tests used to detect infection in the first 72 h according to medical indication.

### 2.3. Statistical Analysis

The data are summarized as absolute and relative frequencies for the qualitative variables. On the other hand, the continuous variables were summarized with measures of central tendency and dispersion according to their distribution. The assumption of normality was assessed using the Shapiro–Wilk test.

### 2.4. DNA Extraction

The Wizard^®^ Genomic DNA Purification Kit (Promega, Madison, Wisconsin, USA) was used to extract and purify complete genomic DNA from 300 μL of blood samples. The extraction process consists of several steps. This included cell lysis, precipitation of proteins and other impurities, DNA fixation with isopropanol, washing with 70% ethanol, and rehydration of the purified DNA. To improve cell lysis, proteinase K (QIAGEN, Hilden, Germany) was added to the lysate with an incubation time of 90 min at 56 °C. The amount and purity of the extracted DNA were evaluated using spectrophotometry. To evaluate DNA integrity, 1% agarose gel electrophoresis (70 V, 70 min) was performed and SYBR™ Safe DNA Gel Stain (Thermo Fisher Scientific, Waltham, MA, USA) was used to visualize the DNA bands.

### 2.5. Sanger Sequencing

Amplification of the V4 region: The V4 region of the 16S rDNA gene was amplified using 515F sense primer (5′-GTGYCAGCMGCCGCGGTAA-3′) and 806R antisense primer (5′-GGACTACNVGGGTWTCTAA-3′). The thermal profile for PCR consisted of the following: initial denaturation at 94 °C for 5 min, followed by 45 cycles of denaturation at 94 °C for 30 s, alignment at 50 °C for 60 s, extension at 72 °C for 5 min, and a final extension at 72 °C for 10 min [17].

Sequencing and analysis: All samples with positive PCR amplicons were sequenced using Sanger sequencing [18]. The obtained sequences were aligned and edited to obtain a consensus sequence using the SeqMan Pro software (DNASTAR, Inc., Madison, WI, USA). Taxonomic classification using the lowest common ancestor (LCM) method was performed using SILVA [19]. Additionally, a search was conducted using the BLASTn bioinformatics tool in the NCBI [20] database for the assignment of species or genera, selecting those with the highest percentage of identity and coverage.

## 3. Results

A total of 28 blood samples were analyzed:  6, 12, and 10 patients in Groups 1 and 2, and 10 in Group 3. The clinical characteristics of the patients are shown in Table 2, and a detailed description of each patient is provided in Supplementary Materials Tabel S1. Using PCR amplification, 19 (67.8%) blood samples were positive for 16S rDNA (*n* = 5 in Group 1, *n* = 8 in Group 2, and *n* = 5 in Group 3). Blood cultures were negative in 100% of the patients.

In Groups 2 and 3, 100% of the mothers and infants received antibiotics during labor and in the first week of life. The median duration of neonatal antibiotic therapy was longer in Group 3 than in Group 2 (7 days [IQR 4.9] vs. 5 days [IQR 2]). The most prescribed antibiotics were ampicillin, gentamicin, piperacillin/tazobactam, cefepime, and linezolid. One patient in Group 3 died on the second day of life due to sepsis, and Pseudomonas mendocina was detected in his blood by molecular testing.

### 3.1. DNA Extraction

The spectrophotometry calculation of the DNA concentration had a median purity of 140.3 ng/μL (IQR 145.3) and measuring the absorbance ratio 260/280 nm yielded a median of 1.84 (IQR 0.084). Integrity was evaluated by performing electrophoresis on a 1% agarose gel, which allowed the observation of medium-high intensity bands without traces of degradation.

### 3.2. Sanger Sequencing

**Figure 1:**
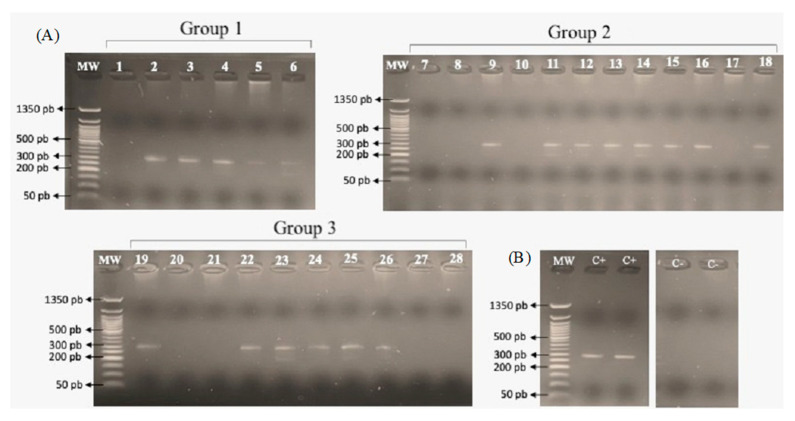
(**A**) Agarose gel showing PCR results to assess amplification of the V4 region of 16S rDNA. The lanes of the PCR gel are marked with the number (1–28) of the case to which the amplification corresponds in each group. (**B**) Agarose gel with results for positive control (bacterial genomic DNA) and negative control (mixture of PCR reagents without the presence of DNA) in duplicate.

Amplicons of approximately 430 bp were generated. Figure 1 shows the PCR results for the 28 neonates. Using SeqMan Pro-11 software (DNASTAR, Madison, WI, USA), the 806R and 515F sequences of each sample were aligned to edit the chromatograms and generate a consensus sequence for identity analyses. After trimming the extremes and before proceeding with manual correction, sequences with overlapping peaks were interpreted as “detected mixed sequences” or ambiguous bases, which were corrected by observing and comparing the peaks, where the competing peak was acceptable when the peak height was greater than the peak of the complementary sequence.

The predominant taxonomic identification in the three groups using BLAST (Basic Local Alignment Search Tool) and SILVA (https://www.arb-silva.de, accessed on 8 September 2023) belonged to the genus Pseudomonas, with identity percentages between 90.2% and 100% (Table 3). Using the BLAST algorithm, eight samples were described as non-cultured bacteria, indicating that this group had not been cultured because of scant information or knowledge about these bacteria. Nine were from the genus Pseudomonas, one was classified as Gammaproteobacteria, and one was a noncultivable organism within the genus Lachnospiraceae. Using the SILVA database, two sequences were not classified, 13 were within the genus Pseudomonas, three were Lachnospiraceae, and one was within the class Clostridia.

## 4. Discussion

Identification of bacteria in the blood with PCR amplification of the variable region V4 of the 16s rDNA gene and Sanger sequencing was performed in preterm neonates with suspected early-onset neonatal sepsis. Blood cultures were negative in all patients, whereas bacteria were identified by PCR V4 16S rDNA in 67.8% of the samples. Interestingly, bacteria were identified in all the patient groups. All analyses were performed under strict asepsis and antisepsis measures to avoid contamination and false positives in PCR results.

Previous studies have shown the usefulness of 16S rDNA PCR-based tests for effective and rapid identification of bacteria during a diagnostic approach to neonatal sepsis [12]. García-Gudiño [21] compared the identification of bacteria by blood culture with PCR of the V3 region of 16S rDNA and denaturing gradient gel electrophoresis (DGGE) in neonates with neonatal sepsis. They observed identification ratios of 40% and 87% between cultures and molecular testing, respectively. However, unlike this study, they failed to separate cases of early- and late-onset sepsis as well as preterm and full-term infants. Likewise, Reier-Nilsen [22], in preterm neonates with suspected infection in the first week of life, compared blood culture with broad-range PCR 16S rDNA, indicating that the latter enhances both sensitivity and specificity in diagnosing confirmed bacterial sepsis. Similarly, other investigations using 16S rDNA PCR targeting specific regions of bacteria related to neonatal sepsis (*Streptococcus agalactiae, Escherichia coli*, and L. *monocytogenes*) have shown better diagnostic performance, especially in culture-negative cases [23,24]. However, research in adults with sepsis using PCR of variable regions of the 16S rDNA and Sanger sequencing has reported the effectiveness of identifying bacteria in patients who have received previous antibiotic treatments and with negative cultures, thus improving stewardship strategies for antibiotic use [25]. The data obtained in this study were consistent with this information. However, it is important to highlight that this study, unlike those previously cited, focused on the analysis of preterm infants with suspected early-onset neonatal sepsis, which was the group that most frequently received antibiotics in the neonatal unit and experienced greater complications owing to unnecessary antibiotic use.

Molecular testing identified bacteria in preterm infants with suspected early-onset sepsis and negative culture results. PCR of the V4 region of 16S rDNA allows for the broader detection of bacteria. This information is important for several reasons. First, this group of patients received most of the antibiotics prescribed in the neonatal unit. Second, false-negative culture results are frequent because of the presence of uncultivated bacteria or difficult-to-isolate organisms and a history of antibiotic use in the mother [26]; the test is especially interesting in the scenario of negative cultures. Third, the test results were available more quickly than when using cultures. This would make it possible to promptly decide on the need for antibiotics, thereby reducing unnecessary exposure to antibiotics and the side effects in this group of patients. However, it is important to recognize that the primers chosen for the variable regions (V1–V9) have an impact on microorganism identification. Although there is no consensus on which V region provides the best results, combining these two regions is recommended. Additionally, it has limitations in terms of diversity resolution and taxonomic analysis [27].

Some bacteria identified in this study were uncultivated bacteria or organisms that were difficult to isolate in traditional cultures. The most frequently identified bacteria were the Pseudomonas spp. Pseudomonas mendocina is a rare cause of infection in humans and has been reported in only 16 adult patients [28]; however, it was identified in the blood of newborns who died during this study. Pseudomonas putida is rarely reported in outbreaks of bacteremia in neonatal units [29]. Conversely, Pseudomonas indoloxydans has not been previously reported to infect humans or neonates and may be a component of the neonatal microbiota [30]. Similarly, although Lachnospiraceae has been identified by PCR of 16S rDNA in amniotic fluid from mothers with neonates who developed early-onset neonatal sepsis [31], it has also been identified as a beneficial microorganism, and its integration into the gut microbiome is necessary for proper neurodevelopment [32].

Regarding the group of preterm infants with no identified risk factors for infection and bacterial DNA, the following considerations were made: The patients required procedures and stabilization in the delivery room, which could have caused transient bacteremia. Likewise, the sociodemographic characteristics of the maternal population treated in the hospital are socially vulnerable from low economic strata, exposed to antibiotic cycles during pregnancy, and carry social risk factors such as malnutrition, which may also explain the transient bacteremia of vertical acquisition.

Regarding the limitations of this study, several considerations are presented. Although, in principle, the identification of bacterial DNA in sterile samples under strict asepsis and antisepsis measures points toward infection, analyses should be carried out to clarify whether bacterial identification is the etiological cause of sepsis [33]. A semiquantitative analysis of the results based on the cycle threshold (Ct) value was proposed, assuming etiological significance when Ct values were low, indicating high pathogen loads [34]. Several statistical approaches have been developed based on the correlation between the relative abundance of samples and the sequencing depth [35]. However, these techniques have limitations in Sanger sequencing [36]; therefore, semiquantitative or diversity analyses were not performed in this study. However, the sample size only allows for descriptive and exploratory analyses of the information, thus preventing the performance of association tests. This initial study of preterm neonates with suspected infection was designed as an exploratory study because of the limitations of research in this specific group of neonates. It is important to recognize the difficulty in recruiting these patients because of their vulnerability. In the future, this study should be scaled up by increasing the sample size to enable the reproduction and generalization of the results.

## 5. Conclusions

In conclusion, PCR V4 16S rDNA allowed the identification of bacteria in the blood of preterm infants with suspected early-onset sepsis and negative cultures. Continued research on variable region PCR and Sanger sequencing in this group of neonates is recommended, as it is a reliable and low-cost method for bacterial identification in neonates with sepsis with a shorter outcome time. Research in this field could allow these tests to be included in neonatal stewardship programs.

## Figures and Tables

**Table 1:** Risk Factors and Criteria for Early Neonatal Infection.

Category	Criteria
Risk Factors for Infection	One or more of the following criteria: -Prolonged rupture of ovulation membranes (PROM) > 18 h. -Chorioamnionitis: fever >38 °C, abdominal tenderness, and paraclinical evidence of systemic inflammatory response. -Preterm birth due to unexplained preterm labor. -In cases of multiple gestations, infection in the other baby.
Early Neonatal Infection: Clinical Sepsis	One or more of the following criteria are present within the first 72 h of life: -Thermal instability: temperature ≤36 °C and/or ≥38 °C. -Heart rate instability with a tendency to tachycardia in the last 24 h: heart rate >180 bpm. -Altered consciousness and/or seizure. -Signs of hemodynamic instability: hypotension (two standard deviations below normal for age), capillary filling > 3 s, cold, mottled, or reticulated skin. -Signs of respiratory instability: tachypnea (respiratory rate > 60 bpm), apnea, increased requirement of inspired fraction of oxygen (FiO_2_) and/or increased requirements for invasive or non-invasive ventilatory support. -Gastrointestinal symptoms due to oral intolerance, abdominal distention. -Laboratory test results: blood count with leukocytes ≥ 22,000 and/or ≤ 4000, platelets ≤ 100,000; immature/total ratio ≥ 0.25; C-reactive protein > 10 mg/dL; and procalcitonin > 10 mg/dL.
Proven early-onset sepsis	Blood cultures identify a microorganism.

**Table 2:** Clinical characteristics of patients.

	Group 1 *n* = 6	Group 2 *n* = 12	Group 3 *n* = 10
Antenatal steroids–*n* (%)			
Yes	5 (83.3)	9 (75)	8 (80.0)
No	1 (16.7)	3 (25)	2 (20.0)
Birth–*n* (%)			
Caesarean section	6 (100)	10 (83,33)	9 (90.0)
Vaginal	0 (0)	2 (16.67)	1 (10.0)
Gestational age–weeks (Ballard) median (IQR1)	31 (1.5)	30.5 (2.5)	29 (3.8)
Sex–*n* (%)			
Male	2 (33.3)	8 (66.67)	8 (80.0)
Female	4 (66.7)	4 (33.33)	2 (20.0)
Birth weight (g) median (IQR)	1440 (411.25)	1220 (327.5)	1120 (686.3)
Leukocyte count (mg/dL) median (IQR)	8990 (2325)	10215 (6948)	7215 (8370)
Absolute neutrophils (mg/dL) median (IQR)	5902.5 (2143.25)	6117 (5352)	5286 (4932.8)
Platelet count (mg/dL) median (IQR)	207,500 (38.500)	224,500 (109.500)	194,000 (64,750)
CRP (mg/dL) median (IQR)	0.7 (0.56)	0.4 (0.225)	0.4 (0.0)
Antibiotic duration (days) median (IQR)	0	5 (2)	7 (4.9)

IQR: Interquartile Range.

**Table 3:** Taxonomic classification results.

Group	ID Patient	BLAST		SILVA	
		Description/Accessions	Identity (%)	LCA* Taxonomy SILVA	Identity (%)
Group 1	2	Uncultured Lachnospiraceae bacterium/KX460604.1	95.83	Clostridia	94.91
	3	Uncultured bacterium/FJ162329.1	90.73	Lachnospiraceae	89.15
	4	Pseudomonas stutzeri/ON514627.1	98.16	Pseudomonas	98.62
	5	Uncultured bacterium/MK494335.1	80.91	Lachnospiraceae	81.99
	6	Uncultured bacterium/MF005955.1	86.88	Unclassified	68.13
Group 2	9	Uncultured bacterium/KC541299.1	90.20	Pseudomonas	95.45
	11	Pseudomonas mendocina/MN276034.1	97.21	Pseudomonas	97.77
	12	Uncultured bacterium/LC683652.1	85.37	Unclassified	75
	13	Gamma proteobacterium/GQ466449.1	96.95	Pseudomonas	97.57
	14	Uncultured bacterium/MH312886.1	96.83	Pseudomonas	94.3
	15	Pseudomonas sp./OR394601.1	99.33	Pseudomonas	100
	16	Pseudomonas sp./MN736123.1	98.38	Pseudomonas	98.92
	18	Pseudomonas indoloxydans/MT435025.1	95.37	Pseudomonas	93.47
Group 3	19	Uncultured bacterium/AM404477.2	94.97	Lachnospiraceae	84.98
	22	Pseudomonas mendocina/MN866768.1	96.14	Pseudomonas	93.96
	23	Uncultured bacterium/KC164827.1	94.24	Pseudomonas	85.34
	24	Pseudomonas indoloxydans/MT435025.1	92.74	Pseudomonas	96.09
	25	Pseudomonas putida/JQ830029.1	100	Pseudomonas	98.83
	26	Pseudomonas sp./CP059139.1	92.83	Pseudomonas	77.08

* LCA: Least common ancestor.

## Data Availability

Raw data supporting the conclusions of this study will be made available by the authors upon request.

## Supplementary Materials

The following supporting information can be downloaded from: https://www.mdpi.com/article/10.3390/tropicalmed9070152/s1, Table S1: clinical description of the neonates.

## References

[1] Popescu C.R., Cavanagh M.M.M., Tembo B., Chiume M., Lufesi N., Goldfarb D.M., Kissoon N., Lavoie P.M. (2020). Neonatal sepsis in low-income countries: Epidemiology, diagnosis and prevention. Expert Rev. Anti-Infect. Ther..

[2] Shane A.L., Sánchez P.J., Stoll B.J. (2017). Neonatal sepsis. Lancet.

[3] Milton R., Gillespie D., Dyer C., Taiyari K., Carvalho M.J., Thomson K., Sands K., Portal E.A.R., Hood K., Ferreira A. (2022). Neonatal sepsis and mortality in low-income and middle-income countries from a facility-based birth cohort: An international multisite prospective observational study. Lancet Glob. Health.

[4] Sola A., Mir R., Lemus L., Fariña D., Ortiz J., Golombek S. (2020). Suspected Neonatal Sepsis: Tenth Clinical Consensus of the Ibero-American Society of Neonatology (SIBEN). Neoreviews.

[5] Mukhopadhyay S., Puopolo K.M. (2017). Clinical and Microbiologic Characteristics of Early-onset Sepsis among Very Low Birth Weight Infants. Pediatr. Infect. Dis. J..

[6] Bedford Russell A.R., Kumar R. (2015). Early onset neonatal sepsis: Diagnostic dilemmas and practical management. Arch. Dis. Child. Fetal Neonatal Ed..

[7] Letouzey M., Lorthe E., Marchand-Martin L., Kayem G., Charlier C., Butin M., Mitha A., Kaminski M., Benhammou V., Ancel P.-Y. (2022). Early Antibiotic Exposure and Adverse Outcomes in Very Preterm Infants at Low Risk of Early-Onset Sepsis: The EPIPAGE-2 Cohort Study. J. Pediatr..

[8] Fleiss N., Schwabenbauer K., Randis T.M., Polin R.A. (2023). What’s new in the management of neonatal early-onset sepsis?. Arch. Dis. Child. Fetal Neonatal Ed..

[9] Meyers J.M., Tulloch J., Brown K., Caserta M.T., D’Angio C.T. (2020). A Quality Improvement Initiative to Optimize Antibiotic Use in a Level 4 NICU. Pediatrics.

[10] Oladokun R.E., Alao M.A., Ogunbosi B.O., Bello O.E., Ude I., Obasi A., Ayede A.I., Tongo O.O. (2023). Trends in Identification, Etiology, and Resistance Profiles of Bacterial Isolates and Appropriate Therapy for Neonatal Sepsis in Low- and Middle-Income Countries: A Narrative Review. Curr. Pediatr. Rep..

[11] Cantey J.B., Lee J.H. (2021). Biomarkers for the Diagnosis of Neonatal Sepsis. Clin. Perinatol..

[12] Wang Y., Zhao J., Yao Y., Yang L., Zhao D., Liu S. (2021). The Accuracy of 16S rRNA Polymerase Chain Reaction for the Diagnosis of Neonatal Sepsis: A Meta-Analysis. BioMed Res. Int..

[13] Hassan R.M., El Enany M.G., Rizk H.H. (2014). Evaluation of broad-range 16S rRNA PCR for the diagnosis of bloodstream infections: Two years of experience. J. Infect. Dev. Ctries..

[14] Mishra D., Satpathy G., Wig N., Fazal F., Ahmed N.H., Panda S.K. (2020). Evaluation of 16S rRNA broad range PCR assay for microbial detection in serum specimens in sepsis patients. J. Infect. Public Health.

[15] Bertolo A., Valido E., Stoyanov J. (2024). Optimized bacterial community characterization through full-length 16S rRNA gene sequencing utilizing MinION nanopore technology. BMC Microbiol..

[16] Yang M.-Q., Wang Z.-J., Zhai C.-B., Chen L.-Q. (2024). Research progress on the application of 16S rRNA gene sequencing and machine learning in forensic microbiome individual identification. Front. Microbiol..

[17] Katiraei S., Anvar Y., Hoving L., Berbée J.F.P., Van Harmelen V., Willems Van Dijk K. (2022). Evaluation of Full-Length versus V4-Region 16S rRNA Sequencing for Phylogenetic Analysis of Mouse Intestinal Microbiota after a Dietary Intervention. Curr. Microbiol..

[18] Sanger F., Nicklen S., Coulson A.R. (1977). DNA sequencing with chain-terminating inhibitors. Proc. Natl. Acad. Sci. USA.

[19] Pruesse E., Quast C., Knittel K., Fuchs B.M., Ludwig W., Peplies J., Glöckner F.O. (2007). SILVA: A comprehensive online resource for quality checked and aligned ribosomal RNA sequence data compatible with ARB. Nucleic Acids Res..

[20] Altschul S.F., Gish W., Miller W., Myers E.W., Lipman D.J. (1990). Basic local alignment search tool. J. Mol. Biol..

[21] García-Gudiño I., Yllescas-Medrano E., Maida-Claros R., Soriano-Becerril D., Díaz N.F., García-López G., Molina-Hernández A., Flores-Herrera O., de la Serna F.J.Z.-D., Peralta-Pérez M.d.R. (2018). Microbiological comparison of blood culture and amplification of 16S rDNA methods in combination with DGGE for detection of neonatal sepsis in blood samples. Eur. J. Pediatr..

[22] Reier-Nilsen T., Farstad T., Nakstad B., Lauvrak V., Steinbakk M. (2009). Comparison of broad range 16S rDNA PCR and conventional blood culture for diagnosis of sepsis in the newborn: A case control study. BMC Pediatr..

[23] Esparcia O., Montemayor M., Ginovart G., Pomar V., Soriano G., Pericas R., Gurgui M., Sulleiro E., Prats G., Navarro F. (2011). Diagnostic accuracy of a 16S ribosomal DNA gene-based molecular technique (RT-PCR, microarray, and sequencing) for bacterial meningitis, early-onset neonatal sepsis, and spontaneous bacterial peritonitis. Diagn. Microbiol. Infect. Dis..

[24] Oeser C., Pond M., Butcher P., Bedford Russell A., Henneke P., Laing K., Planche T., Heath P.T., Harris K. (2020). PCR for the detection of pathogens in neonatal early onset sepsis. PLoS ONE.

[25] Farrell J.J., Hujer A.M., Sampath R., Bonomo R.A. (2015). Salvage microbiology: Opportunities and challenges in the detection of bacterial pathogens following initiation of antimicrobial treatment. Expert Rev. Mol. Diagn..

[26] Celik I.H., Hanna M., Canpolat F.E., Pammi M. (2022). Diagnosis of neonatal sepsis: The past, present and future. Pediatr. Res..

[27] Sune D., Rydberg H., Augustinsson Å.N., Serrander L., Jungeström M.B. (2020). Optimization of 16S rRNA gene analysis for use in the diagnostic clinical microbiology service. J. Microbiol. Methods.

[28] Ioannou P., Vougiouklakis G. (2020). A Systematic Review of Human Infections by Pseudomonas mendocina. Trop. Med. Infect. Dis..

[29] Bouallègue O. (2004). Outbreak of Pseudomonas putida bacteraemia in a neonatal intensive care unit. J. Hosp. Infect..

[30] Manickam N., Ghosh A., Jain R.K., Mayilraj S. (2008). Description of a novel indole-oxidizing bacterium *Pseudomonas indoloxydans* sp. nov., isolated from a pesticide-contaminated site. Syst. Appl. Microbiol..

[31] Wang X., Buhimschi C.S., Temoin S., Bhandari V., Han Y.W., Buhimschi I.A. (2013). Comparative Microbial Analysis of Paired Amniotic Fluid and Cord Blood from Pregnancies Complicated by Preterm Birth and Early-Onset Neonatal Sepsis. PLoS ONE.

[32] Oliphant K., Ali M., D’Souza M., Hughes P.D., Sulakhe D., Wang A.Z., Xie B., Yeasin R., Msall M.E., Andrews B. (2021). *Bacteroidota* and *Lachnospiraceae* integration into the gut microbiome at key time points in early life are linked to infant neurodevelopment. Gut Microbes.

[33] Simner P.J., Miller S., Carroll K.C. (2018). Understanding the Promises and Hurdles of Metagenomic Next-Generation Sequencing as a Diagnostic Tool for Infectious Diseases. Clin. Infect. Dis..

[34] Zautner A.E., Groß U., Emele M.F., Hagen R.M., Frickmann H. (2017). More Pathogenicity or Just More Pathogens?—On the Interpretation Problem of Multiple Pathogen Detections with Diagnostic Multiplex Assays. Front. Microbiol..

[35] Jervis-Bardy J., Leong L.E.X., Marri S., Smith R.J., Choo J.M., Smith-Vaughan H.C., Nosworthy E., Morris P.S., O’Leary S., Rogers G.B. (2015). Deriving accurate microbiota profiles from human samples with low bacterial content through post-sequencing processing of Illumina MiSeq data. Microbiome.

[36] Paul F., Otte J., Schmitt I., Dal Grande F. (2018). Comparing Sanger sequencing and high-throughput metabarcoding for inferring photobiont diversity in lichens. Sci. Rep..

