# Supplementary material for: 16S rDNA Sequencing for Bacterial Identification in Preterm Infants with Suspected Early-Onset Neonatal Sepsis
Source: Trop Med Infect Dis. 2024 Jul 6;9(7):152. doi: 10.3390/tropicalmed9070152 (PMC11281265; doi:10.3390/tropicalmed9070152)
Supplement: Supplementary file 1 [file tropicalmed-09-00152-s001.zip › tropicalmed-3024596-supplementary.pdf]

Table S1. Clinical description of the neonates

ID	Group	Sex	Risk Factors	Clinical course	Paraclinical description	Blood culture	Antibiotics in the mother	Antibiotics in the newborn
1	1	M	14-year-old mother, G1P1. Ballard 31 weeks and emergency abdominal delivery due to eclampsia. Birth weight 1,610 g.	Mechanical ventilation at birth, Pulmonary surfactant upon admission to the neonatal unit, Caffeine citrate therapy, Lactobacillus,	Complete blood count: white blood cells 8100, absolute neutrophils 5792, I/T ratio 0. Platelet count: 182000. PCR: 0.4 mg/dL	N° 1 and 2 negatives	No	No
2	1	F	22-year-old mother, G2P2, 33 weeks Ballard and one dose of antenatal steroid. Emergency abdominal delivery with non-reassuring fetal status and anhydramnios. Birth weight 1,680 g.	High-flow nasal cannula support. Pulmonary surfactant, caffeine citrate therapy, dextrose and amino acid solution. Clinical stability in the first 72 hours.	Complete blood count: white blood cells 11100, absolute neutrophils 7689, I/T ratio 0. Platelet count: 340000. PCR: 0.4 mg/dL	N° 1 and 2 negatives	No	No
3	1	F	30-year-old mother, history of obesity, G2P2, 31 weeks Ballard and single dose of antenatal steroid. Emergency cesarean delivery for HELLP syndrome and fetal bradycardia. Birth weight 1,245 g.	Resuscitation and orotracheal intubation at birth. Presents hemodynamic instability in the first 48 hours, requires initiation of vasopressor medication (dopamine and dobutamine). Receives parenteral nutrition, lactobacillus, and caffeine citrate.	Complete blood count: white blood cells 8690, absolute neutrophils 6013, I/T ratio 0. Platelet count: 198000. PCR: 1.0 mg/dL	N° 1 and 2 negatives	No	No
4	1	F	19-year-old mother, G1P1, twin pregnancy, monochorionic and biamniotic, 35 weeks Ballard, one dose of antenatal steroid and emergency caesarean section for preeclampsia. Birth weight 1,770 g.	Spontaneous neonatal adaptation with clinical stability. Receives amino acids and lactobacillus.	Complete blood count: white blood cells 9290, absolute neutrophils 4905, I/T ratio 0. Platelet count: 193000	N° 1 and 2 negatives	No	No

5	1	F	27-year-old mother, G2P1, 28 weeks Ballard and one dose of antenatal steroid. Emergency cesarean delivery for severe bleeding placenta previa. Birth weight 1,030 g.	Poor general condition at birth, resuscitation and vasopressor support (dopamine). Receives parenteral nutrition, dextrose, caffeine and lactobacillus.	Complete blood count: white blood cells 7010, absolute neutrophils 3435, I/T ratio 0. Platelet count: 238000. PCR: 1.8 mg/dL	N° 1 and 2 negatives	No	No
6	1	M	Madre de 25 años, G1P1, Ballard de 31 semanas, preeclampsia con criterios de gravedad y dos dosis de esteroides antenatales. Nacimiento abdominal de urgencia. Peso al nacer 1.270 g.	Clinical stability, no signs of systemic inflammatory response	Complete blood count: white blood cells 15930, absolute neutrophils 11677, I/T ratio 0. Platelet count: 217000. PCR: 0.7 mg/dL	N° 1 and 2 negatives	No	No
7	2	M	20-year-old mother, G1P1, twin pregnancy (twin 1), 31 weeks Ballard and PROM for 22 hours. Cesarean delivery and preterm birth without cause. Birth weight 1,420 g.	Resuscitation at birth, orotracheal intubation and surfactant deficiency managed with pulmonary surfactant and thrombocytopenia in CBC at 12 hours of life.	Complete blood count: white blood cells 5050, absolute neutrophils 2581, I/T ratio 0. Platelet count: 142000. PCR: 0.4 mg/dL	N° 1 and 2 negatives	No	Ampicillin and gentamicin (3 days)
8	2	M	20-year-old mother, G1P1, twin pregnancy (twin 2), 31 weeks Ballard and PROM for 22 hours. Cesarean delivery. Birth weight 1,560 g.	Resuscitation at birth, orotracheal intubation and surfactant deficiency managed with pulmonary surfactant. Clinical stability.	Complete blood count: white blood cells 5430, absolute neutrophils 2824, I/T ratio 0. Platelet count: 185000. PCR: 0.4 mg/dL	N° 1 and 2 negatives	No	Ampicillin and gentamicin (3 days)
9	2	M	21-year-old mother, G1P1, 28 weeks Ballard, PROM for 48 hours, chorioamnionitis and two doses of antenatal steroids and preterm labor without cause. Emergency cesarean delivery. Birth weight 1,070 g.	Resuscitation, orotracheal intubation at birth and surfactant deficiency managed with pulmonary surfactant.	Complete blood count: white blood cells 20130, absolute neutrophils 15903, I/T ratio 0. Platelet count: 311000. PCR: 0.9 mg/dL	N° 1 and 2 negatives	Erythromycin and Piperacillin/Tazobactam	Ampicillin and gentamicin (5 days)

10	2	M	17-year-old mother, G1P1, active urinary tract infection and 34 weeks Ballard. Preterm without cause and vaginal delivery. Birth weight 1,730 g.	Spontaneous neonatal adaptation.	Complete blood count: white blood cells 12800, absolute neutrophils 8090, I/T ratio 0. Platelet count: 288000.	N° 1 and 2 negatives	Cefazolina	Ampicillin and gentamicin (3 days)
11	2	F	30-year-old mother, G5P5, 33 weeks Ballard, PROM for 19 hours, fetal tachycardia, non-reassuring fetal status and one dose of antenatal corticosteroid. Cesarean delivery and preterm birth without cause. Birth weight 1,080 g.	Respiratory distress and ventilatory support at birth with CPAP and surfactant deficiency managed with pulmonary surfactant. Adequate clinical course in the first 72 hours, without criteria for systemic inflammatory response or sepsis.	Complete blood count: white blood cells 9020, absolute neutrophils 5331, I/T ratio 0. Platelet count: 234000.	N° 1 and 2 negatives	No	Ampicillin and gentamicin (3 days)
12	2	F	39-year-old mother, G4P4, received two doses of antenatal steroid. 31 weeks Ballard, emergency cesarean delivery, preterm without cause, PROM for 29 hours and chorioamnionitis. Birth weight 1,400 g.	Resuscitation and Orotracheal Intubation. Adequate clinical course in the first 72 hours, without criteria for systemic inflammatory response or sepsis.	Complete blood count: white blood cells 12890, absolute neutrophils 8765, I/T ratio 0. Recuento de plaquetas: 292000.	N° 1 and 2 negatives	Erythromycin and Ampicillin	Ampicillin and gentamicin (3 days)
13	2	M	21-year-old mother, G1P1, received two doses of antenatal steroid. 29 weeks Ballard, emergency cesarean delivery, preterm without cause, PROM for 20 hours, anhydramnios and non-reassuring fetal status. Birth weight 1,390 g.	Resuscitation at birth and orotracheal intubation, respiratory distress and requires pulmonary surfactant. Adequate clinical course in the first 72 hours, without criteria for systemic inflammatory response or sepsis.	Complete blood count: white blood cells 11410, absolute neutrophils 6903, I/T ratio 0. Platelet count 254000.	N° 1 and 2 negatives	No	Ampicillin and gentamicin (3 days)
14	2	M	28-year-old mother, G1P1, 32-week Ballard, chorioamnionitis, and antenatal steroid course. Vaginal delivery. Birth weight 1,080 g.	Spontaneous neonatal adaptation. Adequate clinical course in the first 72 hours. No criteria for systemic inflammatory response or sepsis.	Complete blood count: leukocytes 20,200, absolute neutrophils 15,231, I/T ratio: 0. Platelet count: 325,000. PCR 0.4 mg/dL	N° 1 and 2 negatives	Gentamicin and clindamycin	Ampicillin and gentamicin (5 days)

15	2	F	21-year-old mother, G1P1, twin dichorionic, diamniotic pregnancy (twin 2), 29-week Ballard, chorioamnionitis, 24 hours of PROM, and two doses of antenatal steroids. Emergency cesarean delivery and twin 1 with clinical sepsis (case 25). Birth weight 1,115 g.	Resuscitation at birth and orotracheal intubation. Adequate clinical course in the first 72 hours. No criteria for systemic inflammatory response or sepsis.	Complete blood count: leukocytes 6,400, absolute neutrophils 4,685, I/T ratio: 0. Platelet count: 215,000. PCR 0.4 mg/dL	N° 1 and 2 negatives	Ampicillin and erythromycin	Ampicillin and gentamicin (7 days)
16	2	M	25-year-old mother, G2P2, bacterial vaginosis and active urinary tract infection, 30-week Ballard, chorioamnionitis, 46 hours of PPROM, and two doses of antenatal steroids. Abdominal delivery. Birth weight 1,325 g.	Resuscitation at birth and orotracheal intubation. Adequate clinical course in the first 72 hours, without criteria for systemic inflammatory response or sepsis.	Complete blood count: leukocytes 13,750, absolute neutrophils 10,134, I/T ratio: 0. Platelet count: 211,000.	N° 1 and 2 negatives	Ampicillin, erythromycin, cefazolin	Ampicillin and gentamicin (5 days)
17	2	F	26-year-old mother, G2P2, no prenatal care, and regular use of psychoactive substances. 27-week Ballard, two doses of antenatal steroids, preterm without cause, oligohydramnios, non-reassuring fetal status (fetal tachycardia), and cesarean delivery. Birth weight 815 g.	Resuscitation at birth, orotracheal intubation, and surfactant deficiency managed with surfactant replacement therapy. Stable clinical course in the first 72 hours, without criteria for inflammatory response or sepsis.	Complete blood count: leukocytes 6,470, absolute neutrophils 4,031, I/T ratio: 0. Platelet count: 163,000. PCR: 0.7 mg/dL	N° 1 and 2 negatives	No	Ampicillin and gentamicin (7 days)
18	2	M	25-year-old mother, G2, urinary tract infection, and no prenatal care. 26-week Ballard, 20 hours of PROM, two doses of antenatal steroids, preterm without cause, and cesarean delivery. Birth weight 780 g.	Resuscitation at birth and orotracheal intubation, thrombocytopenia, and clinical instability at birth. Stable clinical course in the first 72 hours, without systemic inflammatory response.	Complete blood count: leukocytes 4,980, absolute neutrophils 2,928, I/T ratio: 0. Platelet count: 141,000.	N° 1 and 2 negatives	No	Ampicillin and gentamicin (5 days)

19	3	M	23-year-old mother, G2P2, 27-week Ballard, one dose of antenatal steroid. IUGR, preterm without cause, and cesarean delivery. Birth weight 945 g.	Resuscitation at birth and orotracheal intubation, clinical instability with signs of systemic inflammatory response and hypotension. Complete blood count with leukopenia and thrombocytopenia.	Complete blood count: leukocytes 2,390, absolute neutrophils 1,410, I/T ratio: 0. Platelet count: 104,000. PCR: 0.4 mg/dL	N° 1 and 2 negatives	No	Ampicillin and gentamicin (3 days). Switches to Piperacillin/Tazobactam (10 days)
20	3	M	21-year-old mother, G1P1, 27-week Ballard, and two doses of antenatal steroids. Preterm birth without cause, non-reassuring fetal status, fetal tachycardia, and cesarean delivery. Birth weight 870 g.	Resuscitation at birth, respiratory failure, and orotracheal intubation. Clinical course at birth with systemic inflammatory response, tachycardia, and hypotension requiring vasopressor support.	Complete blood count: leukocytes 15,620, absolute neutrophils 8,747, I/T ratio: 0. Platelet count: 227,000. PCR: 0.4 mg/dL	N° 1 and 2 negatives	No	Ampicillin and gentamicin (7 days)
21	3	M	26-year-old mother, suspected congenital toxoplasmosis, G3P3, 31-week Ballard, 20 hours of PROM, and two doses of antenatal steroids. Preterm without cause and vaginal delivery. Birth weight 1,600 g.	Resuscitation at birth and orotracheal intubation. Clinical deterioration in the first 48 hours due to increased ventilator respiratory parameters, control blood count with thrombocytopenia, neutrophilia, and positive PCR, chest X-ray with pneumonia. In utero pneumonia is considered. Early sepsis.	Complete blood count: leukocytes 8,250, absolute neutrophils 5,288, I/T ratio: 0.2. Platelet count: 193,000. PCR: 11.4 mg/dL	N° 1 and 2 negatives	Spiramycin	Ampicillin and gentamicin (1.5 days). Switch to cefepime and ampicillin (10 days)
22	3	F	15-year-old mother, G1P1, and no prenatal care. 28-week Ballard, 36 hours of PROM, no antenatal steroids, and preterm without cause. Cesarean delivery and birth weight 700 g.	Resuscitation at birth and orotracheal intubation. Systemic inflammatory response with tachycardia, bradycardia, shock, and hypotension requiring vasopressor management. Death.	Complete blood count: leukocytes 2,420, absolute neutrophils 1,120, I/T ratio: 0.3. Platelet count: 158,000. Procalcitonin 14.3 mg/dL	N° 1 and 2 negatives	Ampicillin and gentamicin	Ampicillin and gentamicin (2 days)

23	3	M	34-year-old mother, G3P3, with active urinary tract infection and two doses of antenatal steroids. 31-week Ballard, preterm without cause, and emergency cesarean delivery. Birth weight 1,500 g.	Resuscitation at birth, orotracheal intubation, respiratory deterioration in the first 24 hours of life, surfactant deficiency managed with surfactant replacement therapy, and chest X-ray with in utero pneumonia. Early clinical sepsis.	Complete blood count: leukocytes 6,360, absolute neutrophils 3,218, I/T ratio: 0. Platelet count: 194,000. PCR: 0.4 mg/dL	N° 1 and 2 negatives	Cephalexin	Ampicillin and gentamicin (7 days)
24	3	M	21-year-old mother, G1P1, dichorionic, diamniotic twin pregnancy (twin 1), 29-week Ballard, chorioamnionitis, 24 hours of PROM, and two doses of antenatal steroids. Emergency cesarean delivery and twin 2 at risk for early infection (case 15). Birth weight 1,100 g.	Resuscitation at birth, orotracheal intubation, clinical deterioration at 48 hours, surfactant deficiency managed with surfactant replacement therapy, and increased ventilator parameters. Chest X-ray with in utero pneumonia.	Complete blood count: leukocytes 5,090, absolute neutrophils 2,855, I/T ratio: 0. Platelet count: 235,000. PCR: 0.4 mg/dL	N° 1 and 2 negatives	Ampicillin and erythromycin	Ampicillin and gentamicin (2 days), switch to Piperacillin/Tazobactam (7 days) then to Ampicillin (7 days)
25	3	M	22-year-old mother, G1P1, 25-week Ballard, 80 hours of PROM, chorioamnionitis, two doses of antenatal steroids, and non-reassuring fetal status (fetal tachycardia). Cesarean delivery and birth weight 820 g.	Resuscitation at birth, orotracheal intubation, surfactant deficiency managed with surfactant replacement therapy, respiratory failure, and clinical instability with signs of systemic inflammatory response given by shock requiring vasopressor medication, and in utero pneumonia.	Complete blood count: leukocytes 8,250, absolute neutrophils 5,288, I/T ratio: 0.2. Platelet count: 193,000. PCR: 11.4 mg/dL	N° 1 and 2 negatives	Ampicillin and erythromycin	Ampicillin and gentamicin (5 days), switch to cefepime/linezolid completing 7 days
26	3	M	20-year-old mother, G3C2P1, only prenatal control, urinary tract infection, and chorioamnionitis. 33-week Ballard, two doses of antenatal steroids. Cesarean delivery and birth weight 1,745 g.	Hemodynamic instability in the first hours of life, with shock and requiring vasopressor medication. Spontaneous neonatal adaptation.	Complete blood count: leukocytes 17,320, absolute neutrophils 11,656, I/T ratio: 0.2. Platelet count: 263,000.	N° 1 and 2 negatives	Clindamycin and gentamicin	Ampicillin and gentamicin (7 days)

27	3	F	20-year-old mother, G3C2P1, only prenatal control, urinary tract infection, and chorioamnionitis. 33-week Ballard, two doses of antenatal steroids. Cesarean delivery and birth weight 1,745 g.	Hemodynamic instability in the first hours of life, with shock and requiring vasopressor medication. Spontaneous neonatal adaptation.	Complete blood count: leukocytes 17,320, absolute neutrophils 11,656, I/T ratio: 0.2. Platelet count: 263,000.	N° 1 and 2 negatives	Ampicillin and gentamicin	Ampicillin and gentamicin (7 days)
28	3	M	20-year-old mother, G2C2, no prenatal care, 33-week Ballard, preterm labor, 20% abruption, and one dose of antenatal steroid. Non-reassuring fetal status (fetal tachycardia). Cesarean delivery and birth weight 1,785 g.	Spontaneous neonatal adaptation, without requiring support at birth. Deterioration at 36 hours with apnea and respiratory deterioration, increased FiO2 and need for orotracheal intubation.	Complete blood count: leukocytes 7,260, absolute neutrophils 4,305, I/T ratio: 0.2. Platelet count: 258,000. PCR 0.4 mg/dL	N° 1 and 2 negatives	No	Onset at 36 hours with ampicillin and gentamicin (7 days)

Group 1, patients without suspected early-onset sepsis; **Group 2**, patients with suspected early neonatal infection and no clinical signs of early neonatal infection. **Group 3**, patients with suspected early neonatal infection and clinical infection in the first 72 hours of life. F: Female; M: male; G: Number of pregnancies; C: Number of cesarean deliveries; P: Number of vaginal deliveries; PROM: Prolonged rupture of membranes; I/T: Immature/Total neutrophil ratio; PCR: C-reactive protein; FiO2: Fraction of inspired oxygen
